# Development of a Ternary Solid Dispersion Formulation of LW6 to Improve the In Vivo Activity as a BCRP Inhibitor: Preparation and In Vitro/In Vivo Characterization

**DOI:** 10.3390/pharmaceutics11050206

**Published:** 2019-05-01

**Authors:** Rajiv Bajracharya, Sang Hoon Lee, Jae Geun Song, Minkyoung Kim, Kyeong Lee, Hyo-Kyung Han

**Affiliations:** College of Pharmacy, Dongguk University-Seoul, Dongguk-ro-32, Ilsan-Donggu, Goyang, Korea; rajivbajra@hotmail.com (R.B.); sh_lee@dongguk.edu (S.H.L.); dkfmrhtm@gmail.com (J.G.S.); kyoung2k@naver.com (M.K.); kaylee@dongguk.edu (K.L.)

**Keywords:** LW6, solid dispersion, BCRP inhibitor, topotecan, bioavailability

## Abstract

LW6 (3-[2-(4-adamantan-1-yl-phenoxy)-acetylamino]-4-hydroxy-benzoic acid methyl ester) is a potent inhibitor of drug efflux by the breast cancer resistance protein (BCRP). However, its poor aqueous solubility leads to low bioavailability, which currently limits in vivo applications. Therefore, the present study aimed to develop ternary solid dispersion (SD) formulations in order to enhance the aqueous solubility and dissolution rate of LW6. Various SDs of LW6 were prepared using a solvent evaporation method with different drug/excipient ratios. The solubility and dissolution profiles of LW6 in different SDs were examined, and F8-SD which is composed of LW6, poloxamer 407, and povidone K30 at a weight ratio of 1:5:8 was selected as the optimal SD. The structural characteristics of F8-SD were also examined using Fourier transform infrared spectroscopy (FTIR), differential scanning calorimetry (DSC), X-ray powder diffraction (XRPD), and scanning electron microscopy (SEM). In the acidic to neutral pH range, F8-SD achieved rapid dissolution with a drug release of 76–81% within 20 min, while the dissolution of pure LW6 was negligible. The XRPD patterns indicated that F8-SD probably enhanced the solubility and dissolution of LW6 by changing the drug crystallinity to an amorphous state, in addition to the solubilizing effect of the hydrophilic carriers. Furthermore, F8-SD significantly improved the oral bioavailability of topotecan, which is a BCRP substrate, in rats. The systemic exposure of topotecan was enhanced approximately 10-fold by the concurrent use of F8-SD. In conclusion, the ternary SD formulation of LW6 with povidone K30 and poloxamer 407 appeared to be effective at improving the dissolution and in vivo effects of LW6 as a BCRP inhibitor.

## 1. Introduction

The breast cancer resistance protein (BCRP), also designated as ABCG2, is the second member of the G subfamily of the ATP binding cassette (ABC) efflux transporters, which restricts the systemic exposure and organ distribution of various exogenous compounds, including drugs and toxins [1,2]. It is ubiquitously expressed in normal tissues including the small intestine, liver, kidney, blood–brain barrier, testis, placenta, and mammary glands, in addition to cancer cells [1]. BCRP substrates comprise a variety of therapeutic and nontherapeutic compounds, including topotecan, irinotecan, mitoxantrone, methotrexate, cimetidine, nitrofurantoin, and dietary flavonoids [2]. Accordingly, BCRP has a significant impact on the pharmacokinetics and drug resistance of various drugs. Thus, the Food and Drug Administration (FDA) and European Medicines Agency guidelines endorse the assessment of the interaction potential of new drug candidates with BCRP during the new drug discovery and development process [3]. Given that BCRP plays a key role in reducing the bioavailability of various drugs and also developing the multidrug resistance of anticancer drugs, a potent BCRP inhibitor may be valuable in improving the effectiveness of drug therapy. 

**Figure 1:**
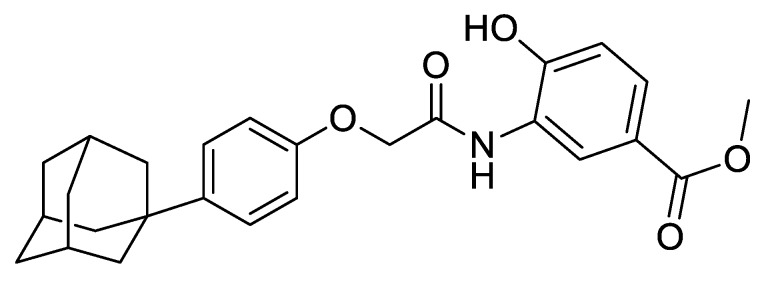
Structure of LW6 (3-[2-(4-adamantan-1-yl-phenoxy)-acetylamino]-4-hydroxy-benzoic acid methyl ester).

LW6 (3-[2-(4-adamantan-1-yl-phenoxy)-acetylamino]-4-hydroxy-benzoic acid methyl ester, Figure 1) has been identified as a potent inhibitor of BCRP, resulting in an enhanced cellular uptake of BCRP substrates [4]. In previous reports, LW6 was found to be even more potent than Kol43, which is a typical BCRP inhibitor. Furthermore, as a hypoxia-inducible factor 1α (HIF-1α) inhibitor, LW6 showed a significant increase in the intracellular reactive oxygen species (ROS) levels, inducing cytochrome C release as a result of mitochondrial dysfunction, leading to apoptosis [5,6]. Considering that 90% of metastatic cancer patients developed chemotherapy resistance leading to failure in the treatment [7], combined regimens of LW6 with anticancer drugs may provide synergistic chemotherapy effects via BCRP inhibition, as well as its own anticancer effect. However, due to its highly lipophilic nature, LW6 is poorly water-soluble, and its resulting low bioavailability limits the in vivo applications. Therefore, there is a strong demand for an effective formulation that improves the solubility and in vivo effect of LW6. 

There are various pharmaceutical approaches for enhancing solubility, including salt formation, particle size reduction, solid dispersion, the self-nanoemulsifying drug delivery system, and lipid-based drug delivery systems [8,9,10,11,12,13,14]. Among them, the preparation of solid dispersion (SD) with hydrophilic polymers is one of the widely applicable, simple, and cost-effective methods [12,15]. SD can be prepared by hot-melt extrusion, solvent evaporation, or spray-drying methods [16,17]. In SDs, the poorly water-soluble drugs are molecularly dispersed in hydrophilic polymers such as polyvinylpyrrolidone, polyethylene glycol, hydroxypropyl methylcellulose, and poloxamer to improve their dissolution profile [18]. Since hydrophilic polymers may help prevent the recrystallization of amorphous drugs in supersaturation conditions, the selection of an optimal polymer is crucial for the development of effective SD formulations [18,19]. Furthermore, depending on the desired dissolution profiles, stability, and drug properties, additional excipients such as surfactant or adsorbent may often be incorporated into ternary SDs [20,21]. Vojinović et al. [20] showed an improved release of carbamazepine by ternary SDs using a hydrophilic polymer and a surface adsorbent. Since surfactants improve the wettability and physical stability of SDs [21], the selection of an appropriate surfactant also plays a vital role in SD preparation.

Therefore, in the present study, ternary SDs of LW6 were prepared using hydrophilic carriers and surfactants at various drug–polymer ratios. In vitro characterization was performed to select the optimal SD formulation for improving the solubility and dissolution of LW6. In vivo effects of the SD formulation on the bioavailability of BCRP substrates were also examined using topotecan, which is an archetypal BCRP substrate in rats.

## 2. Materials and Methods

### 2.1. Materials

LW6 was a kind gift from Prof. Kyeong Lee (Dongguk University, Goyang, Korea). Polyethylene glycol 6000, poloxamer 188 (Kolliphor^®^ P188), poloxamer 407 (Kolliphor^®^ P407), povidone K12 (Kollidon^®^12 PF, PVP K12), and povidone K30 (Kollidon^®^30, PVP K30) were obtained from BASF-Korea (Seoul, Korea). Hydroxypropyl methyl cellulose E5 (HPMC E5) was obtained from Colorcon Asia Pacific PTE LTD. (Suwon, Korea). Span-20, Span-80, sodium dodecyl sulfate (SDS), and topotecan were purchased from Sigma Aldrich (St. Louis, MO, USA). All the other chemicals were of analytical grade, and all the solvents were of high-performance liquid chromatography (HPLC) grade.

### 2.2. Screening of Excipients and Preparation of SDs

For the selection of optimal carriers, SDs of LW6 were prepared with various hydrophilic polymers at a drug–carrier ratio of 1:5 using the solvent evaporation method. Briefly, LW6 was dissolved in dichloromethane by sonication for 10 min for complete solubilization. Each polymeric carrier was dissolved in dichloromethane and added to the drug solution. After vigorous mixing, all the solvents were eliminated under vacuum at room temperature. The resulting product was milled and sieved through an 80-mesh screen. The corresponding physical mixtures (PMs) were also prepared by mixing LW6 and each carrier with a mortar and pestle. The carrier that enhanced the LW6 solubility to the greatest extent was selected for preparing the ternary SD formulations of LW6. The effect of surfactants on the solubility of LW6 was also determined using a 10% aqueous solution of various surfactants.

**Figure 2:**
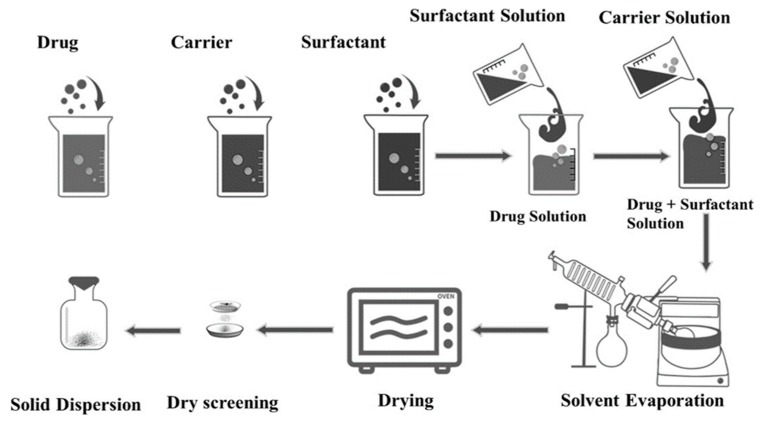
Preparation of ternary solid dispersions.

Once the optimal carrier and surfactant were selected, ternary SDs and PMs were prepared at various weight ratios of each component, as illustrated in Figure 2.

### 2.3. Solubility Studies

Each formulation equivalent to 1 mg of LW6 was dissolved in 1 mL of distilled water and stirred at 300 rpm for 48 h at room temperature. The samples were centrifuged at 13,000 rpm for 10 min, and the supernatant was filtered through an 0.45-µm pore-sized cellulose syringe filter (Target^®^, National scientific, Mansfield, TX, USA). The drug concentration in each filtrate was measured by HPLC assay.

### 2.4. Structural and Morphological Characterizations of SDs

Fourier transform infrared spectroscopy (FTIR) spectra were obtained using an ATR-FTIR spectrophotometer (Nicolet™ iSTM 5, Thermo-Fisher Scientific, Waltham, MA, USA) with a ZnSe crystal accessory. The spectrum of each sample was collected over the wavenumber range of 4000 to 400 cm^−1^ with 32 scans at a resolution of 4 cm^−1^.

The thermal transition properties of samples were assessed using differential scanning calorimetry (DSC Q2000, TA instruments, Ghent, Belgium) equipped with an intercooler. Indium was used to calibrate the temperature and heat flow. The samples were placed in aluminum pans and heated from 25 °C to 300 °C at a scanning rate of 10 °C/min under an inert atmosphere flushed with nitrogen gas at a flow rate of 30 mL/min.

X-ray powder diffraction (XRPD) patterns were examined at room temperature using an X-ray diffractometer (X’Pert APD, PHILIPS, Amsterdam, The Netherlands). The samples were placed on a zero-background silicon holder. The diffraction pattern was measured with CuKα radiation at 40 kV and 30 mA over a 2*θ* range of 3° to 60° using a step size of 0.02° at a scan speed of 1 s/step. The XRPD study was conducted at the Korea Basic Science Institute (Daegu Center, Korea).

The morphology of the pure drug, polymeric carriers, and SDs were examined using a field emission scanning electron microscope (FE-SEM). The samples were spread on a specimen stub using double-sided sticky tape, coated with platinum, and examined by a scanning electron microscope (SU-70, Hitachi, Tokyo, Japan) at an acceleration voltage of 20 kV.

### 2.5. In Vitro Drug Release Studies

The drug release profiles of each formulation were examined at different pH conditions (1.2, 4.0, and 6.8). Each formulation (drug amount equivalent to 1 mg of LW6) was dispersed in different release media (10 mL) and stirred at 100 rpm, 37 °C. At predetermined time points (10, 20, 30, 45, and 60 min), the samples were collected and filtered through a 0.45-µm pore-sized cellulose syringe filters. The drug concentration in each filtrate was determined by HPLC assay.

### 2.6. Pharmacokinetic Studies in Rats

The pharmacokinetic profiles of topotecan, which is a representative BCRP substrate, were examined in rats with/without the co-administration of LW6 in different formulations. Animal studies were carried out in accordance with the “Guiding Principles in the Use of Animals in Toxicology” adopted by the Society of Toxicology (USA), and the study protocol was approved by the review committee of Dongguk University (IACUC-2017-016-2). Male Sprague–Dawley rats (260–280 g) were provided by Orient bio Co., Ltd. (Seongnam, Korea). All the rats were given free access to tap water and a normal standard chow diet (Superfeed Company, Wonju, Korea). The rats were fasted for 18 h before the experiments, and divided into three groups (n = 3 per group). The pure drug or SD (equivalent to 20 mg/kg of LW6) was suspended in 0.5% aqueous methylcellulose with 5% polyethylene glycol (PEG) and topotecan (10 mg/kg) was dissolved in saline. Each formulation was given orally to the three groups of rats (group 1, topotecan only; group 2, topotecan + pure LW6; group 3, topotecan + SD of LW6). Blood samples were obtained from the femoral artery at the predetermined time points. The blood samples were centrifuged at 13,000 rpm for 5 min, and the obtained plasma samples were frozen at −20 °C until analyzed by liquid chromatography-tandem mass spectrometry (LC-MS/MS).

### 2.7. Analytical Methods

*HPLC assay for LW6:* The concentrations of LW6 from in vitro samples were determined using a HPLC system (Perkin Elmer series 200, Waltham, MA, USA) consisting of a UV detector, a pump, and an automatic injector. A reversed-phase C18 column (Gemini C18, 4.6 × 150 mm, 5 µm; Phenomenex, Torrance, CA, USA) was eluted with a mobile phase consisting of acetonitrile and 0.1% formic acid (80:20, v/v). The flow rate was 1.0 mL/min at 30 °C, and the UV wavelength was set at 254 nm. Valsartan was used as an internal standard, and the calibration curve of LW6 was linear (*r*^2^ = 0.99) within the concentration range of 0.05 to 100 µg/mL.

*LC-MS/MS assay for topotecan:* The concentration of topotecan in rat plasma was determined using LC-MS/MS assay as reported by Ye et al. [22]. Briefly, 100 µL of plasma, 20 µL of the internal standard camptothecin (1 µg/mL), and 180 µL of methanol were mixed vigorously, and then centrifuged at 13,000 rpm for 5 min. After centrifugation, 240 µL of the supernatant was collected and dried under vacuum. After complete drying, the residue was re-dispersed in methanol and analyzed by LC-MS/MS. Chromatographic separation was done with a C18 column (4.6 × 100 mm, 2.6 μm; Phenomenex, Torrance, CA, USA) using a mobile phase consisting of acetonitrile and 0.1% formic acid (80:20, v/v) at a flow rate of 0.4 mL/min. Mass spectrometric detection was done using the AB Sciex API 4000 triple quadrupole mass spectrometer (AB Sciex, Framingham, MA, USA). The electrospray ionization (ESI) source was set in positive ionization mode. The precursor/product ion pair (m/z) was 422.2/377.1 for topotecan and 349.1/305.2 for camptothecin (IS). The calibration curve was prepared at concentrations of 1 to 1000 ng/mL with a good linearity of *r*^2^ value greater than 0.99. 

### 2.8. Pharmacokinetic and Statistical Analysis

Based on the noncompartmental analysis, the area under the plasma concentration–time curve (AUC) was calculated using the linear trapezoidal method. The peak plasma concentration (Cmax) and the time to reach the peak plasma concentration (Tmax) were values recorded from the experimental data.

The data were represented as mean values with standard deviation. Statistical analysis was performed using one-way ANOVA followed by Dunnett’s test. A *p*-value < 0.05 was considered statistically significant.

## 3. Results and Discussions

### 3.1. Selection of Excipients

Since LW6 has the high melting point (279 °C), the SDs of LW6 were prepared with various polymers at a weight ratio of 1:5 using the solvent evaporation method. Then, the aqueous solubility of each SD formulation was determined and compared to that of pure LW6 for the selection of an optimal carrier. Since LW6 was practically insoluble in water due to its hydrophobic nature (Clog P of 6.49 ± 0.42), its aqueous solubility was predicted as 0.00264 µg/mL using an online database [23]. As summarized in Table 1, among the tested carriers, povidone K30 (PVP K30) enhanced LW6 solubility to the greatest extent, which was approximately 7200-fold higher than the predicted solubility of LW6. This may be due to an improved wettability as well as the inhibition of crystal growth in the presence of PVP K30. Previous studies have also suggested that PVP K30 could effectively inhibit recrystallization and maintain the amorphous form of drugs in SDs [24,25]. Furthermore, PVP K30 exhibits a favorable profile with low toxicity, high water solubility, and biocompatibility [26]. Therefore, PVP K30 was selected as an optimal carrier for the SD formulation of LW6.

**Figure 3:**
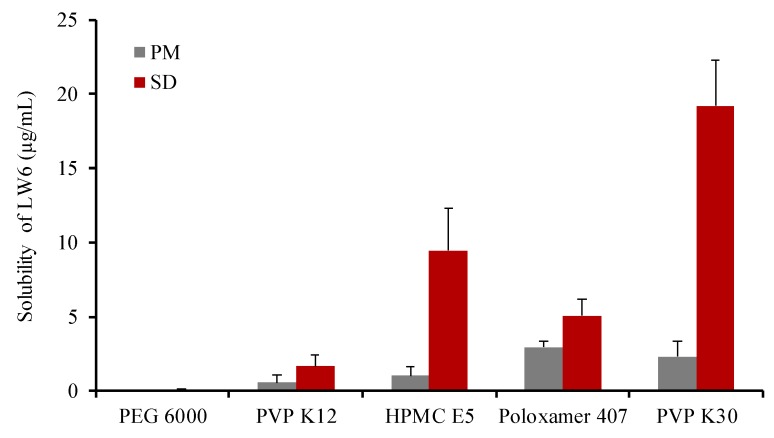
The apparent solubility of LW6 in SDs and physical mixtures (PMs) with different polymers. Data are expressed as the mean ± standard deviation (n = 3).

In comparison with the corresponding PMs, the SD formulations produced significantly (p < 0.05) higher enhancement in the solubility of LW6 (Figure 3), implying that the change in drug crystallinity to an amorphous state may also contribute to the enhanced solubility by SDs in addition to the solubilizing effect of hydrophilic carriers. Therefore, the drug crystallinity was examined as described in Section 3.3.

**Figure 4:**
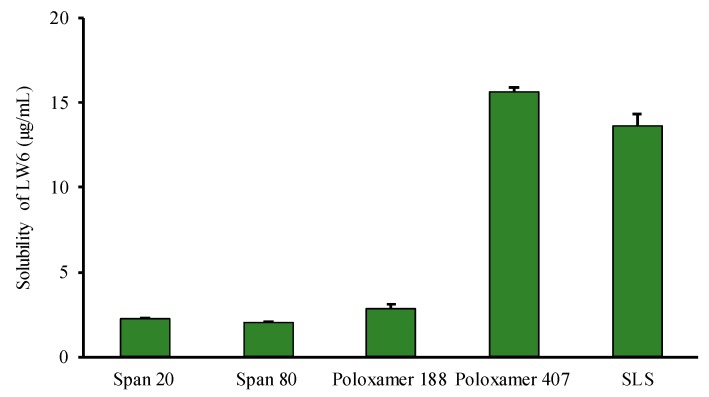
Effect of surfactants on the solubility of LW6. Data are expressed as the mean ± standard deviation (n = 3).

Since surfactants improve the wettability and physical stability of SDs [21], the selection of an appropriate surfactant also plays a vital role in SD preparation. Therefore, the effect of surfactants on the solubility of LW6 was examined using various surfactants. Among the tested surfactants, poloxamer 407 was most effective at enhancing the solubility of LW6 (Figure 4). Since poloxamer 407, a nonionic surfactant, is a triblock copolymer consisting of a hydrophobic block of polypropylene glycol and two hydrophilic blocks of polyethylene glycol (PEG), it has amphiphilic properties. Therefore, it can form micelles and distribute hydrophobic drugs into the core of the micelles, resulting in improved drug solubility [27]. Moreover, poloxamer 407 can demonstrate a dual function of the surface-active agent and polymeric carrier in SDs, and also exhibits a favorable oral safety profile [27,28]. As the use of a polymer blend may have a synergistic effect on enhancing the stability and dissolution of amorphous drugs in ternary dispersion systems [29], in the present study, ternary SDs of LW6 were prepared using a blend of PVP K30 and poloxamer 407.

### 3.2. Optimization of SD Formulations

**Figure 5:**
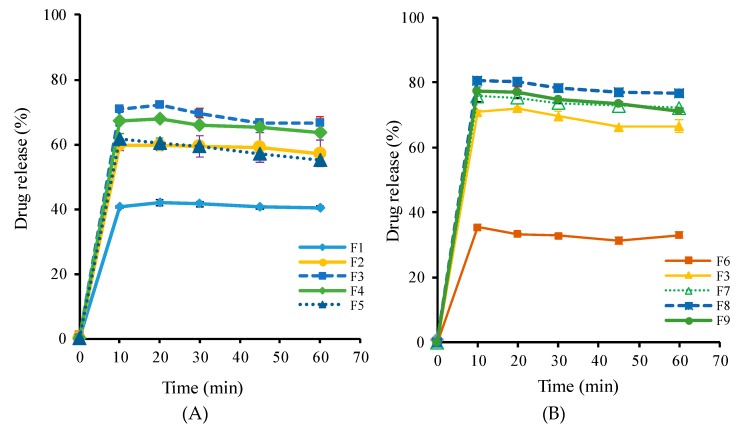
Drug release profiles of various SD formulations in water. Data are expressed as the mean ± standard deviation (n = 3). (**A**) Effect of poloxamer 407; (**B**) Effect of PVP K30.

Since the binary SD formulations with PVP K30 or poloxamer 407 at a 1:5 drug–excipient ratio exhibited the poor and slow drug release of less than 5% within 1 h, the present study attempted to develop the ternary SD formulations. Single-factor analysis was used to optimize the composition of ternary SD formulations. Various SD formulations (F1–F9) were prepared using different drug–excipient ratios as summarized in Table 2; then, the drug release studies were carried out in water. As illustrated in Figure 5, depending on the ratio of PVP K30 and poloxamer 407, all the tested SDs exhibited a rapid drug release of 35–81% within 1 h. Both PVP K30 and poloxamer 407 showed similar effects on drug release from SDs—that is, the extent of drug release increased as the weight ratio of PVP K30 or poloxamer increased (Figure 5). This could be explained by several factors, including the increased wettability of the drug particles, micellar solubilization, and the inhibition of recrystallization in the presence of hydrophilic carriers [24,30]. However, the solubilization effect of excipients reached its maximum at the drug–polymer ratio of 1:5:8, and a further increase in the proportion of each excipient had a negative impact on the drug release (Figure 5). This could be explained at least in part by the reversible gellation properties of poloxamer 407. As reported by Fakhari et al. [31], the increase in poloxamer 407 concentration increased the viscosity due to higher physical entanglement, which retards the diffusion and release of the drug from the polymeric matrix. Similarly, at a higher ratio of PVP K30, the solubilization effect may be neutralized by the diffusion process due to the increased viscosity of the solution around the solid particles [32]. Thus, the drug release does not increase proportionally with the amount of PVP K30. Consequently, the F8 formulation was selected as an optimized SD formulation for further characterization.

**Figure 6:**
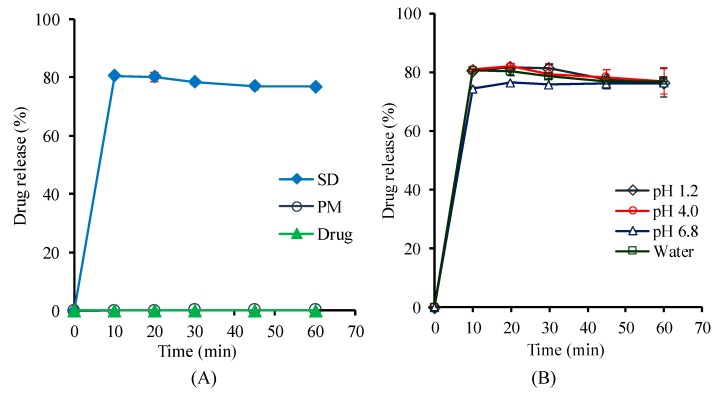
Drug release profiles of the optimal SD formulation (F8-SD) in water. Data are expressed as the mean ± standard deviation (n = 3). (**A**) Comparison of SD, PM, and pure drug; (**B**) drug release profiles of F8-SD at different pHs.

The drug release profiles of the selected SD formulation (F8-SD) were evaluated in comparison with those of the pure drug and its corresponding PM. As shown in Figure 6A, the SD formulation significantly enhanced the dissolution rate, as well as the extent of drug release, exhibiting approximately 80% drug release within 1 h, while the drug dissolution from pure drug and its corresponding PM was minimal. This result could be explained by several factors, including improved drug wetting and micellar solubilization in the presence of hydrophilic carriers, as well as the amorphous state of the drug in the SD formulation. Furthermore, the F8-SD exhibited a similarly high and fast drug release in pH conditions ranging from acidic to neutral, suggesting an effective drug release during the transition along the gastrointestinal tract (Figure 6B).

### 3.3. Structural and Morphological Characterization of SD Formulation

The structural and morphological characterization of the drug in SDs were performed using DSC, XRPD, FT-IR, and SEM as described below.

#### 3.3.1. Differential Scanning Calorimetry (DSC)

**Figure 7:**
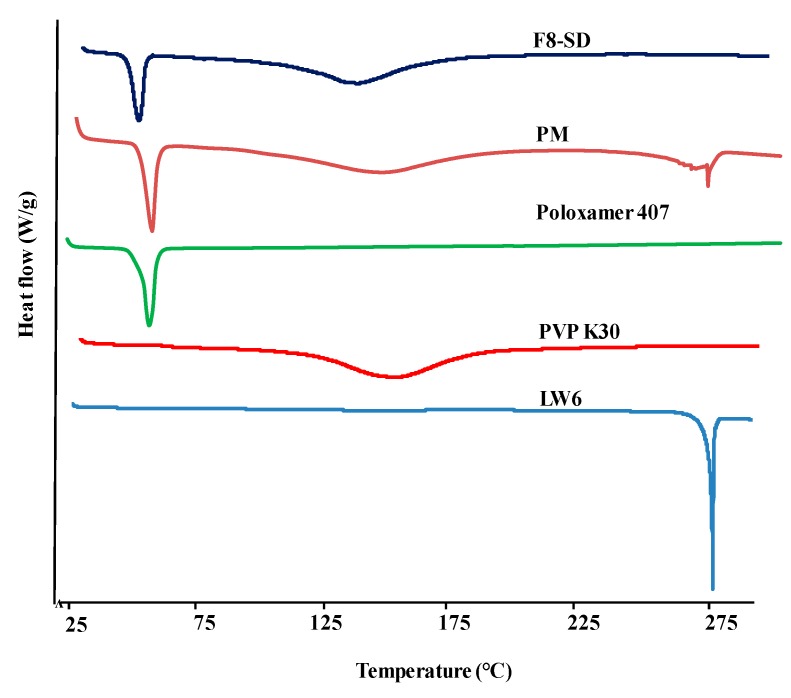
Differential scanning calorimetry (DSC) thermograms of drug, polymeric carriers, F8-SD, and PM.

The DSC thermograms of pure LW6, PVP K30, poloxamer 407, F8-SD, and PM were obtained to examine the characteristics of thermal behaviors. As illustrated in Figure 7, LW6 exhibited a single and sharp endothermic peak at 279 °C, indicating its melting point. This high melting point also suggests its highly crystalline nature. Likewise, the thermogram of hygroscopic PVP K30 showed a wide endothermic peak at 150.57 °C due to the dehydration of polymer, while poloxamer 407 had a sharp peak at 55.55 °C; these are comparable to the values reported in the literature [24,33]. The characteristic endothermic peaks of the drug and each excipient were observed in PM, implying that the crystal nature of the drug was retained in the physical mixture. 

In contrast, the F8-SD formulation showed an absence of the melting endothermic peak of LW6 (Figure 7). This disappearance of the melting endothermic peak of the drug in SD formulation may be due to a change in the crystallinity of the drug to amorphous form, producing a eutectic mixture, or the solubilization of LW6 in the molten carrier during thermal analysis. In addition, the presence of two exothermic peaks at 48.01 °C and 132.61 °C in the SD formulation indicates that the polymers themselves had either partially or fully separated from each other due to polarity and conformational flexibility issues. 

#### 3.3.2. X-ray Powder Diffraction (XRPD) Studies

**Figure 8:**
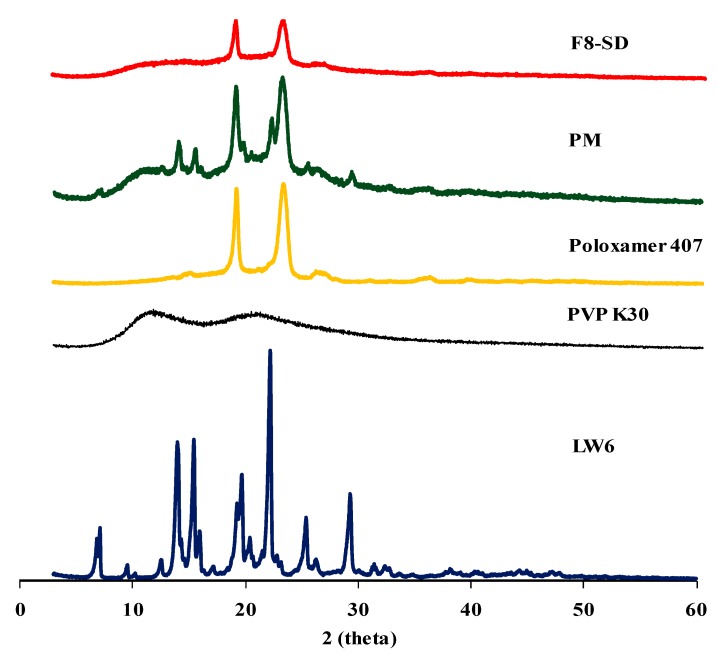
X-ray powder diffraction (XRPD) diffractograms of LW6, polymeric carriers, PM, and F8-SD.

XRPD analysis was used to examine the drug crystallinity in each formulation. The XRPD patterns of pure LW6, polymeric carriers, PM, and F8-SD are summarized in Figure 8. Pure LW6 exhibited multiple distinct peaks at 7.15°, 14.01°, 15.47°, 19.71°, 22.23°, 25.41°, and 29.33°, indicating the crystalline nature of the drug. PM also exhibited some distinct peaks similar to the pure drug, implying that the crystallinity of LW6 did not change in the physical mixture. In addition, the characteristic peaks of poloxamer 407 were observed at 18.99° and 23.09° in PM. According to previous studies, poloxamer 407 is semi-crystalline in nature, and shows a peak around 19° and 23° of 2*θ* [34], whereas PVP K30 is amorphous, lacking diffraction peaks [24]. While PM exhibited some distinct peaks from the pure drug and poloxamer 407, the preparation of the SD formulation resulted in the disappearance of the distinct peaks of crystalline LW6, while the diffraction peaks of poloxamer 407 were retained. These results suggest that the drug crystallinity was changed to an amorphous form in SD formulation. Therefore, in addition to the micelle-solubilizing effect of the hydrophilic carriers, the amorphous state of LW6 may also contribute to the enhanced solubility of LW6 by SD formulation. 

#### 3.3.3. FT-IR

**Figure 9:**
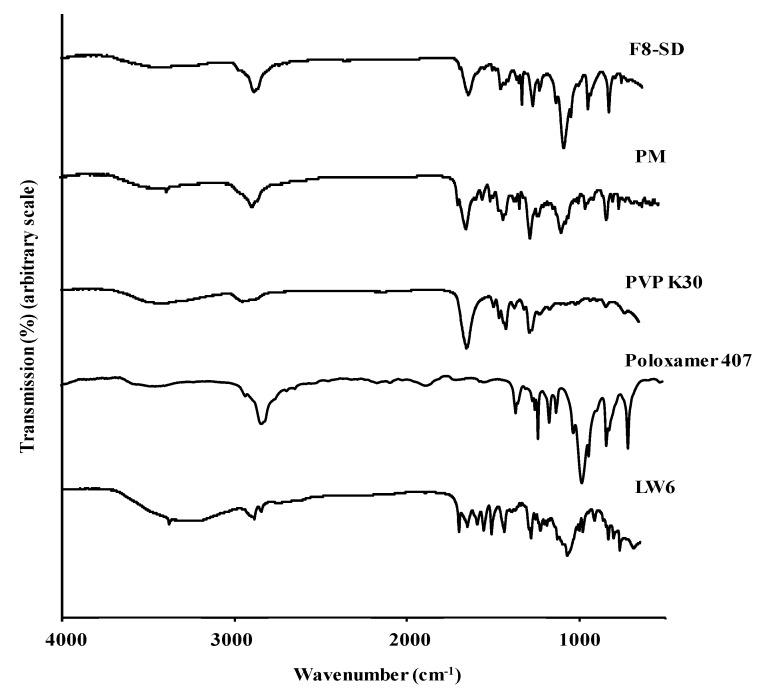
Fourier transform infrared (FT-IR) spectra of LW6, F8-SD, PM, and polymeric carriers.

FT-IR analysis was used to examine the drug–polymer interaction in ternary SDs. As shown in Figure 9, poloxamer 407 showed absorption bands at 3470 cm^−1^ (–OH stretching), 2879 cm^−1^ (aliphatic –CH stretching), 1465 cm^−1^ (–CH_2_ bending), and 1097 cm^−1^ (C–O stretching vibration). The FT-IR spectrum of PVP K30 exhibited a broad band at 3434 cm^−1^ from the OH stretching vibrations of absorbed water, confirming the broad endotherm detected in the DSC study. The most distinct peak was the stretching vibration of the carbonyl group at 1651 cm^−1^. In the case of LW6, the broad peak from the –OH stretching was observed at ~3500 to 3100 cm^−1^ along with a small sharp peak at 3379 cm^−1^ from the –NH vibration. 

Compared to the absorption bands from each component, the FT-IR spectrum of PM showed a superposition of peaks from the drug and excipients, suggesting that there were no intermolecular interactions between them, and LW6 may retain its crystallinity in PM. In contrast, the disappearance of certain peaks and band shifts were observed in SD formulation as compared to those from the drug and individual excipient. The shift in the broad peak of –OH stretching and the disappearance of the –NH vibration peak indicated an interaction between the drug and the excipients. The disappearance of the carbonyl group peak of LW6 at 1698 cm^−1^ also reflects the formation of a hydrogen bond with a proton-donating site of polymers. The interaction of the drug with polymeric carriers, as well as the decreased mobility of LW6 in the hydrophilic matrix, could promote the stable amorphous state of the drug in SDs.

#### 3.3.4. Scanning Electron Microscopy (SEM)

**Figure 10:**
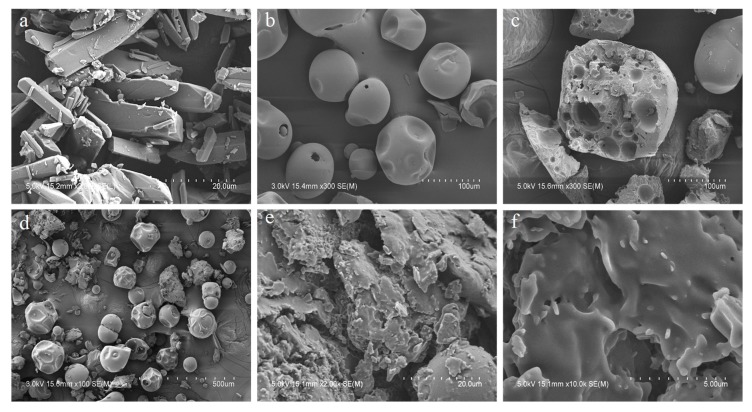
SEM images of LW6 (**a**); PVP K30 (**b**); Poloxamer 407 (**c**); PM (**d**); and F8-SD (**e**, **f**). Scale bar: 20 µm (**a**, **e**); 100 µm (**b**, **c**); 500 µm, (**d**); and 5 µm (**f**).

The morphological characteristics of the SD formulation, polymeric carriers, and the pure drug were examined by SEM. As shown in Figure 10, LW6 showed bar-shaped crystal structures, but SD exhibited a homogeneous blend of all the ternary components in irregular-shaped particles, indicating the amorphous state of the drug.

### 3.4. Pharmacokinetic Studies

Topotecan is a topoisomerase inhibitor that is used as the second-line treatment of patients with metastatic ovarian carcinoma and small cell lung cancer [34]. Given that the oral absorption of topotecan is strongly limited by intestinal BCRP [1], the coadministration of LW6, which is a BCRP inhibitor, may improve the oral absorption of topotecan. Therefore, in the present study, the in vivo effects of LW6 in SD formulation (F8-SD) on the oral pharmacokinetics of topotecan were examined in rats.

**Figure 11:**
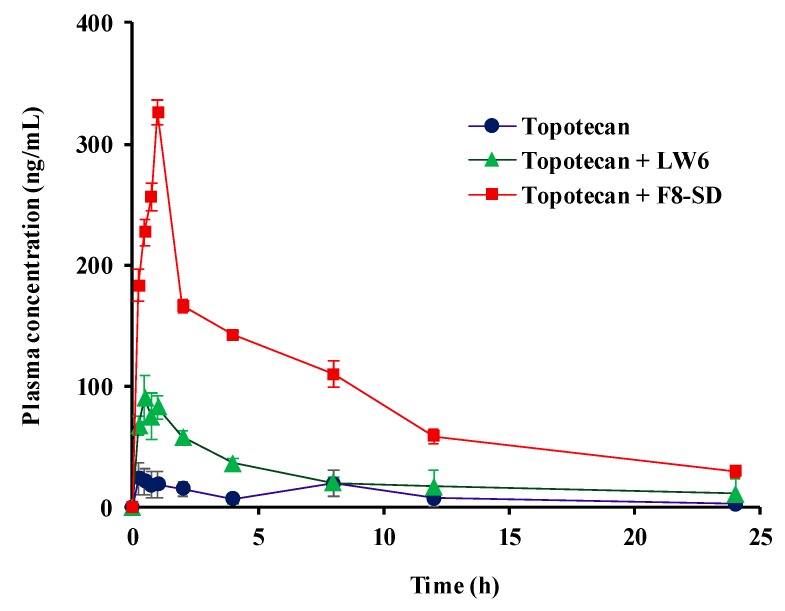
Plasma concentration–time profiles of topotecan following the oral administration of topotecan (10 mg/kg) to rats in the presence or absence of LW6 (20 mg/kg) in different formulations. Data are expressed as the mean ± standard deviation (n = 3).

The pharmacokinetic parameters and the plasma concentration–time profiles of topotecan are summarized in Table 3 and Figure 11. The concurrent use of LW6, particularly in SD formulation, significantly improved the oral absorption of topotecan in rats. The coadministration of pure LW6 or F8-SD with topotecan increased the oral exposure of topotecan by approximately threefold and 10-fold, respectively (Table 3). Considering that topotecan is a BCRP substrate limiting the oral absorption and the affinity of topotecan for P-gp is low [1,2], these results may be attributed to the effective inhibition of BCRP-mediated drug efflux by LW6 during the intestinal absorption of topotecan. Furthermore, the enhanced solubility and dissolution of F8-SD could increase the luminal concentration of LW6, resulting in the much greater inhibition of BCRP-mediated drug efflux compared to pure LW6. The potential contribution of BCRP inhibition by excipient itself cannot be excluded at this point, since some of the pharmaceutical excipients exhibit the inhibition effect on BCRP-mediated drug efflux [35,36]. Taken together, the developed SD formulation of LW6 appeared to be effective at improving the oral exposure of the BCRP substrate, topotecan, in rats.

## 4. Conclusions

The ternary SD formulation of LW6 with PVP K30 and poloxamer 407 appeared to be effective at improving the dissolution and in vivo effectiveness of poorly soluble LW6.

## Figures and Tables

**Table 1:** Apparent aqueous solubility of LW6 in solid dispersions (SDs) with different polymers. Data are expressed as the mean ± standard deviation (n = 3). PEG: polyethylene glycol, PVP: povidone, HPMC: Hydroxypropyl methyl cellulose.

Carriers	Solubility (µg/mL)
PEG 6000	0.057 ± 0.078
PVP K12	1.659 ± 0.729
HPMC E5	9.470 ± 2.874
Poloxamer 407	5.057 ± 1.105
PVP K30	19.20 ± 3.112

**Table 2:** Composition of ternary SDs at the different weight ratios (w/w).

Group	Formulation	Weight Ratio (w/w/w)		
		LW6	PVP K30	Poloxamer 407
	F1	1	5	1
	F2	1	5	3
1	F3	1	5	5
	F4	1	5	6
	F5	1	5	7
	F6	1	1	5
	F3	1	5	5
2	F7	1	7	5
	F8	1	8	5
	F9	1	9	5

**Table 3:** Pharmacokinetic parameters of topotecan following the oral administration of topotecan (10 mg/kg) in the presence and the absence of LW6 (20 mg/kg) in different formulations to rats. Data are expressed as the mean ± standard deviation (n = 3). AUC: area under the plasma concentration–time curve.

Parameters	Topotecan	Topotecan + LW6	Topotecan + F8-SD
C_max_ (ng/mL)	31.3 ± 7.1	91.5 ± 17.7 *	326 ± 25.2 *
T_max_ (h)	0.4 ± 0.1	0.4 ± 0.1	1.0 *
AUC (ng*h/mL)	204 ± 11.1	598 ± 188 *	2140 ± 46.6 *
T_1/2_ (h)	2.0 ± 0.4	9.3 ± 4.8 *	8.8 ± 1.5 *

* p < 0.05, compared to the control given topotecan only.
